# The gut-retina axis in age-related macular degeneration: immune crosstalk and metabolite production

**DOI:** 10.3389/ebm.2026.10847

**Published:** 2026-02-23

**Authors:** Beryl Zhou, Zaid Parekh, Christopher Phung, Sarah H. Rodriguez, Dimitra Skondra

**Affiliations:** 1 Pritzker School of Medicine, University of Chicago, Chicago, IL, United States; 2 Department of Ophthalmology and Visual Science, University of Chicago, Chicago, IL, United States; 3 NYU Langone Health, New York, NY, United States

**Keywords:** age-related macular degeneration, bile acids, complement system, gut microbiome, gut-retina axis, immune crosstalk, short-chain fatty acids

## Abstract

Current therapies slow down advanced features but do not halt or reverse degeneration and neovascularization in dry and wet age-related macular degeneration (AMD). Recent research implicates the gastrointestinal microbiome as a potential critical modulator in AMD pathogenesis through the gut-retina axis. Dysbiosis, characterized by imbalanced microbial diversity, composition and function, can exacerbate systemic and retinal inflammation through microglial priming, inflammasome activation, and secretion of pro-angiogenic cytokines (IL-6, IL-1β, TNF-α, VEGF). Additionally, microbiome-derived metabolites such as short-chain fatty acids and bile acids may exert modulatory roles in host immunity and homeostasis. Their depletion in conjunction with enrichment of specific microbial taxa have been linked to progression of advanced AMD. Together, these complex systems of immune crosstalk in relation to dysbiosis highlight the gut-retina axis as a promising therapeutic target. Dietary modifications, particularly Mediterranean and high-fiber diets, enhance production of protective metabolites and are associated with decreased AMD progression risk compared to Western dietary patterns. Experimental strategies such as fecal microbiota transplantation in animal models and drug repurposing strategies show promise in modulating disease severity. This review synthesizes current mechanistic insights into microbial-immune crosstalk in AMD, emphasizing the interplay of dysbiosis, immune activation, and metabolite signaling.

## Impact statement

This review summarizes emerging evidence connecting the gut microbiome with age-related macular degeneration (AMD), focusing on immune crosstalk and the influence of microbial metabolites on retinal health. Integrating recent findings, we discuss how changes in gut microbial composition, including shifts in short-chain fatty acids and bile acids, may contribute to chronic inflammation and complement dysregulation in the retina. We highlight how the interplay between genetic risk, diet, and the gut microbiome shapes AMD risk and progression. By framing AMD within a systemic context, this review points to the gut-retina axis as a relevant target for future interventions. These insights offer a clearer rationale for microbiome-based strategies in AMD care, supporting ongoing efforts to translate basic research into more effective, holistic approaches to prevention and treatment.

## Introduction

Age-related macular degeneration (AMD) is a progressive, multifactorial disease that remains the leading cause of irreversible vision loss among older persons/people in developed countries [1]. It is characterized by the accumulation of extracellular deposits (drusen) beneath the retinal layer, impairing nutrient exchange and causing degeneration of the retinal pigment epithelium (RPE) and overlying photoreceptors in the macula, the region responsible for central vision [1, 2]. AMD is generally categorized into early, intermediate, and advanced stages based on retinal changes [3]. Advanced AMD presents as either dry AMD—progressive degeneration of RPE and retina that results in geographic atrophy—or wet AMD, defined by choroidal neovascularization (CNV) with abnormal blood vessel growth, hemorrhage, and fluid leakage [3, 4].

Current strategies can slow disease progression, such as antioxidant supplementation (AREDS2) for early AMD and vascular endothelial growth factor (VEGF) inhibitors for wet AMD, but no treatment can cure or reverse the disease [5–7]. As a multifactorial disorder, several modifiable environmental factors such as smoking, obesity, and a high-fat diet have consistently been associated with both the onset and progression of AMD [8, 9]. However, the biological pathways underlying these associations remain unclear. Emerging evidence suggests that the gut microbiome may serve as a link between lifestyle factors and AMD pathogenesis.

The complex community of microorganisms in the gastrointestinal tract plays a central role in systemic immune homeostasis, and its disruption (dysbiosis) has been linked to numerous inflammatory and neurodegenerative disorders [10–13]. In mouse models and *in vitro* studies, changes in microbiota composition can exacerbate retinal degeneration by modulating cytokine profiles, altering the availability of short-chain fatty acids (SCFA) and bile acids (BA), and activating host immune pathways [14]. It is important to note that in addition to gut microbes, a low-density but increasingly recognized ocular surface microbiome has also been described on the conjunctiva and corneal surface [15]. Although its composition is sparse compared with the gut, perturbations in ocular surface communities have been linked to dry eye disease and other inflammatory ocular surface disorders, suggesting that local microbial signals contribute to barrier function and immune tone at the front of the eye [15]. However, while this recent work has summarized a bidirectional ‘gut–eye surface axis,’ this review synthesizes current knowledge on the gut–retina axis in AMD specifically, with a focus on proposed mechanistic links, key immune pathways, and prospects for microbiome-based interventions.

## Altered gut microbiome in AMD

The human gut microbiota is comprised of six major phyla: Bacillota (formerly Firmicutes), Bacteroidota (formerly Bacteroidetes), Actinomycetota (formerly Actinobacteria), Fusobacteriota, Verrucomicrobiota, and Pseudomonadota (formerly Proteobacteria) [16]. A healthy microbiome, characterized by a balanced microbial composition, helps maintain gut barrier integrity and suppress inflammation by reducing translocation of endotoxins such as lipopolysaccharides (LPS) from Gram-negative bacteria [17]. High levels of serum LPS have been found with diabetic microvascular complications, indicating its potential pathological role in retinal diseases [18, 19]. In contrast, dysbiosis represents disruption or reduced diversity in microbial composition, leading to increased intestinal permeability, endotoxemia, and systemic immune activation [20].

Patients with AMD exhibit distinct gut bacterial signatures compared to those without AMD, which can be further modulated by dietary glycemic/high-fat burden. In general, AMD patients share a gut microbiome profile shifted towards higher levels of inflammatory bacteria and decreased levels of beneficial SCFA-producers [21]. Andriessen et al. demonstrated that high-fat diets in mouse models enriched Clostridia (a class of bacteria within Firmicutes phylum) and Proteobacteria, and was associated with an increase in CNV [22]. Alternatively, Rowan et al. showed that mice on high-glycemic diets were more likely to develop AMD-associated phenotypes including RPE atrophy and photoreceptor degeneration, with similar Clostridia enrichment [23]. Early work on human subjects by Zinkernagel et al. also found that wet AMD patients had gut microbiomes with distinct increases in bacteria from the Clostridia class (*Anaerotruncus, Oscillibacter, Ruminococcus torques, Eubacterium ventriosum*) and a depletion of the beneficial *Bacteroides eggerthii* compared to controls [24]. As *Bacteroides* are major producers of SCFAs, this depletion may promote a pro-inflammatory state conducive to neovascularization [24, 25]. In wet AMD, there is an overrepresentation of mucin-degrading (*R. torques)* and Gram-negative taxa (e.g., Negativicutes, *Desulfovibrio*), which are associated with increased LPS and angiogenic signals. Although these findings were observational, these changes could specifically enhance VEGF-driven vascular growth that can lead to wet AMD.

Subsequent studies have confirmed and extended these findings. Using shotgun metagenomics, Zysset-Burri et al. observed overrepresentation of the class Negativicutes in wet AMD patients while Lin et al. found increased relative abundance of *Prevotella* and *Desulfovibrio* in advanced AMD patients [11, 23, 26, 27]. Notably, *Prevotella*’s role in inflammation is context-dependent, often seen as a beneficial marker in fiber metabolism, yet also associated with chronic inflammatory diseases such as rheumatoid arthritis in Western diets [26, 28, 29]. *Desulfovibrio*, a sulfate-reducing bacterium, is recognized for generating hydrogen sulfide, which also has context-dependent effects in ocular tissues [30, 31]. At physiologic levels, endogenous hydrogen sulfide produced by ocular enzymes participates in vascular regulation and antioxidant defense pathways that can support retinal cell survival [32, 33]. However, excessive or prolonged exposure leads to oxidative injury and cell death in corneal and retinal pigment epithelium models, underscoring how dysregulated sulfide production may contribute to retinal degeneration [34].

Although several studies have characterized gut microbial alterations in wet AMD, direct comparisons of microbiome profiles across the spectrum of AMD stages remains limited. Parekh et al. found that stool samples of advanced AMD patients had significant increased abundance of several taxa within the pro-inflammatory *Clostridia* class compared to intermediate AMD and controls [14]. While both dry and wet AMD share features of reduced gut microbial diversity and depletion of anti-inflammatory SCFA producers, wet AMD has notable associations with the enrichment of LPS-producing bacteria [23, 27, 35, 36]. This endotoxin can trigger Toll-like receptor 4 (TLR4) inflammatory pathways and has also been implicated in neurodegenerative diseases such as Huntington’s disease, Alzheimer’s disease, and amyotrophic lateral sclerosis [31].

Despite these findings, the temporal relationship between microbial changes and clinical AMD progression is poorly understood, with most studies utilizing cross-sectional samples that limit the ability to infer causality. Ongoing studies are addressing this gap by investigating how administering probiotics in mouse models may influence disease progression via CNV size and drusen deposition [37].

## Microbial metabolites: short-chain fatty acids and bile acids

Alterations in the host microbiome can also induce signaling via microbial-derived metabolites, synthesized by microbiota through the digestion of dietary products. SCFAs such as acetate, propionate, and butyrate are key microbial metabolites produced through the fermentation of indigestible carbohydrates, which regulate lipid metabolism and immune homeostasis [38]. Although there are no single producers of each SCFA, studies have shown that Bacteroidetes phylum predominantly produce propionate, whereas Firmicutes phylum—particularly the families *Lachnospiraceae* and *Ruminococcaceae*—produce high levels of butyrate [27, 39].

The protective effects of SCFAs are well-documented. In general, SCFAs are associated with a reduced risk of inflammatory bowel disease (IBD), obesity, and allergic airway inflammation [40]. Mechanistically, oral administration of acetate in mice showed a GPR43-mediated decrease in peripheral neutrophil activation alongside reduced surface expression of pro-inflammatory receptors in models of colitis, arthritis, and asthma [39]. In mouse models of uveitis, propionate suppressed Th1 and Th17 frequency and reduced effector T cell migration from the intestine, while promoting regulatory T lymphocytes for immune homeostasis [41]. In the context of AMD, it is believed that these fatty acids may reach the retina through the blood-retinal barrier and act via G-protein coupled receptors to induce protective effects on the RPE and microglia [42, 43].

Bile acids (BAs) have also emerged as important mediators in the pathogenesis of AMD with their influence extending beyond systemic lipid metabolism to retinal health [44]. Although primary BAs are synthesized in the liver, they are secondarily modified by gut microbes and thus dysbiosis may alter levels of protective BAs. Two secondary bile acids, ursodeoxycholic acid (UDCA) and tauroursodeoxycholic acid (TUDCA) are protective against photoreceptor degeneration, diabetic retinopathy (DR), and laser-induced CNV in several models of retinal disease [45]. In a mouse model of prolonged light exposure, TUDCA has been shown to reduce accumulation of superoxide radicals in the outer retina and preserve photoreceptors from apoptosis [46]. Taurocholic acid (TCA) also offers protective effects for tight junction integrity of RPE cells *in vitro*, and inhibited VEGF-induced angiogenesis in choroidal endothelial cells [47]. Many of these effects are mediated by farnesoid X receptor (FXR) and Takeda G-protein receptor 5 (TGR5), which regulate various mediators of endothelial cell dysfunction in diabetic retinopathy [48]. Glycocholic acid and glycoursodeoxycholic acid (GUDCA) biosynthesis pathways have also been found to be decreased in the plasma of wet AMD patients [49, 50]. In the eye, alternative extrahepatic production by enzymes such as CYP27A1 and CYP46A1 enables local BA synthesis critical for cholesterol clearance and ocular function [51]. Deficiency of these enzymes in *Cyp27a1* ^*−/−*^ *Cyp46a1* ^*−/−*^ knockout mice resulted in AMD-like phenotypes, including neovascularization and retinal macrophage activation [52].

A pilot study evaluating stool samples of control, intermediate AMD, and advanced AMD patients found distinct differences in both SCFAs and BAs between groups [14]. Advanced AMD patients had a decreased abundance of key protective SCFAs including acetate, butyrate, and propionate, as well as BAs such as TCA and TUDCA. Notably, functional annotation analysis indicated differential expression of genes related to fermentation and carbohydrate metabolism pathways. Although a mechanistic link could not be determined, this underscores a broader role for microbial dysregulation in inhibiting key enzymatic pathways for SCFA and BA production while promoting an inflammatory state that may exacerbate retinal pathologies.

## Innate immunity, cytokines, and complement

**FIGURE 1:**
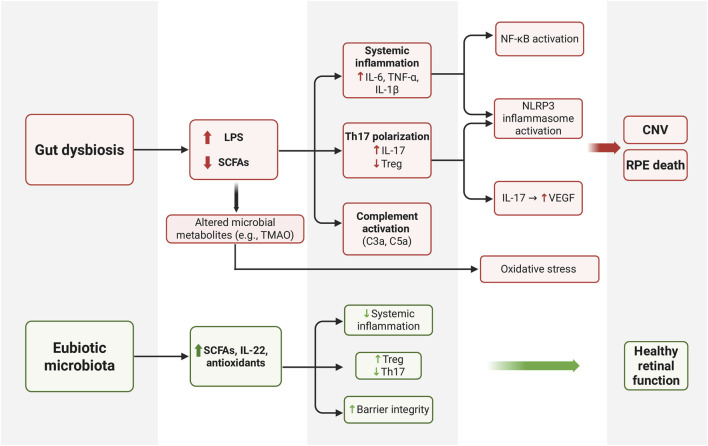
Immune signaling linking gut dysbiosis to retinal degeneration. Gut dysbiosis increases LPS and alters microbial metabolites which promotes systemic inflammation, Th17 polarization, and complement activation (C3a, C5a). These changes cause oxidative stress and inflammasome signaling which lead to retinal cell injury. Maintenance of a healthy microbiome promotes anti-inflammatory metabolites, regulatory T cell balance, and healthy retinal function. LPS, lipopolysaccharide; SCFA, short-chain fatty acid; RPE, retinal pigment epithelium; CNV, choroidal neovascularization.

In addition to peripheral signaling through metabolites, the interplay of the gut microbiome and the innate-immune system represents a central mechanistic axis linking its involvement in AMD. Specifically, these pathways involve the complement cascade, inflammatory cytokines, and tissue-resident immune cells of the retina (Figure 1) [53].

Alterations in the complement cascade are strongly linked to AMD pathogenesis. In healthy eyes, complement factor H (CFH) normally acts to suppress the complement activation, preventing accelerated lipoprotein accumulation that contributes to drusen formation [54]. Notably, a particular genetic variant of CFH (Y402H) has been implicated in 35%–63% of AMD cases and is suggested to significantly elevate AMD risk by impairing CFH’s ability to bind heparan sulfate on Bruch’s membrane [55–59]. This can lead to uncontrolled alternative complement pathway activation, permitting deposition of pro-inflammatory complement components such as C3b and the membrane attack complex (C5b-9) in subretinal tissues [60]. In dry AMD, these deposits can damage the RPE and drive geographic atrophy (GA), whereas in wet AMD they stimulate VEGF secretion and promote CNV [61, 62]. Additionally, recent microbiome profiling studies have found that AMD patients exhibit enrichment of Gram-negative taxa with LPS (e.g., *Negativicutes* and *Escherichia-Shigella*) alongside high-risk CFH alleles implicated in complement dysregulation [35, 62]. One proposed mechanism is that increased circulating bacterial antigens in the gut and peripheral tissues sequester CFH away from retinal targets, exacerbating retinal inflammation and drusen formation [63]. This highlights the complement system as a key mechanistic link within the interplay of host genetics and specific bacterial communities.

In addition to host genetic variants, specific gut microbes can also directly modulate complement activation at the intestinal mucosa. Recent work by Wu et al. demonstrated that microbiota-induced local complement production in the gut shapes both pathogen clearance and tolerance of commensal bacteria, with distinct community structures associated with higher or lower complement activity [64]. These findings support the concept that dysbiosis in AMD may not only increase circulating microbial antigens but also shift gut complement tone toward a more pro-inflammatory state, thereby amplifying systemic complement activation that could exacerbate retinal injury.

Gut-derived LPS and other pathogen-associated molecular patterns (PAMPs) can also prime retinal microglia and choroidal macrophages, leading to inflammasome activation and cytokine signaling [18]. Under stress or injury, innate immune cells in the retina and choroid transition from surveillant to reactive phenotypes. In early AMD, microglia migrate from the inner retina to subretinal spaces where they accumulate around drusen and damaged RPE cells [65, 66]. Human histological studies reveal increased densities of IBA1+ and HLA-DR+ myeloid cells in the submacula of early AMD, while experimental mouse models demonstrate that activated microglia and infiltrating macrophages secrete pro-inflammatory cytokines and matrix metalloproteinases (MMPs) that degrade tissue and promote vascularization [67–69]. AMD patients also exhibit enrichment of CD4^+^ Th1 and Th17 cells that promote the polarization of monocytes toward inflammatory M1 macrophage states while inducing secretion of IFN-γ and IL-17 [70].

Both mouse models and human histological studies indicate that activation of the NLRP3 inflammasome within choroidal macrophages and RPE is central to the chronic inflammatory milieu observed in AMD. This multimeric complex governs IL-1β production, which requires two signals for activation [71]. First, a priming step involves NF-κB activation, commonly from systemic LPS binding to Toll-like receptor (TLR4) activation [72, 73]. Secondly, the trigger step usually involves danger signals such as oxidized LDL and amyloid-β 1-40, which are abundant in drusen formations [74]. Upon entry into the eye, the monocytes that were primed by LPS then become highly responsive to local stimuli such as drusen and rapidly release IL-1β that damages the RPE. These pro-inflammatory signals further induce RPE cells to enhance leukocyte recruitment, perpetuating a positive-feedback loop [59].

The causal role of the gut microbiome in modulating these immune responses has been further supported by dysbiosis studies and fecal microbiota transplantation (FMT) experiments. In the laser-induced mouse model of CNV, high-fat diet-induced obesity was shown to exacerbate lesions through gut microbiota-driven mechanisms [22]. The resulting dysbiosis increased intestinal permeability and systemic release of pro-inflammatory cytokines interleukin (IL)-6, IL-1β, tumor necrosis factor (TNF-α) as well as ocular VEGF-A, thereby promoting pathological angiogenesis.

## Future directions for the gut–retina axis

**FIGURE 2:**
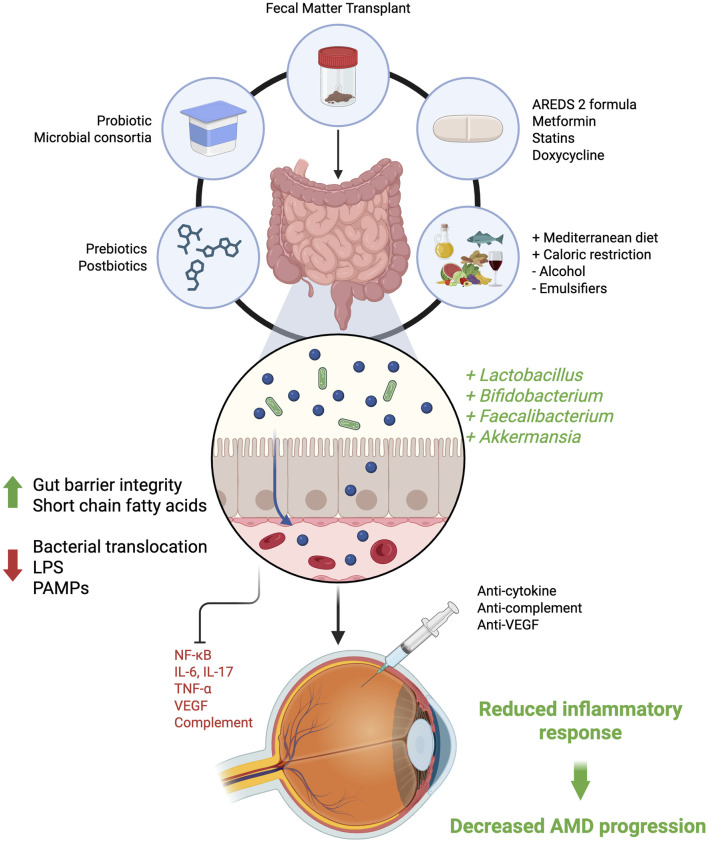
Modifying the gut microbiome as therapy for AMD. Interventions that target the gut microbiome such as FMT, probiotics, dietary changes, and selected medications work to promote beneficial bacteria, enhance gut barrier integrity, and reduce gut inflammatory mediators; together slowing AMD progression. LPS, lipopolysaccharide; FMT, fecal microbiota transplantation; SCFA, short-chain fatty acids; AMD, age-related macular degeneration; PAMPs, pathogen-associated molecular patterns; VEGF, vascular endothelial growth factor.

Given the direct role that diet has on gut microbiome composition and its known role as a modifiable risk factor for AMD, future studies can better assess specific diets that may maximize the presence of protective microbes in AMD patients (Figure 2) [75, 76]. The Age-Related Eye Disease Study (AREDS) was a multicenter, randomized, placebo-controlled trial that discovered that supplementation with vitamin C, E, zinc, copper, lutein, and zeaxanthin reduced the risk of progression to advanced AMD [77–79]. While the original AREDS and AREDS2 trials did not directly measure gut microbiome composition, the micronutrient formulations they tested are now recognized to influence oxidative stress and systemic inflammatory tone, factors that can shape gut microbial communities. More recently, a randomized clinical trial of micronutrient supplementation (lutein, zeaxanthin, vitamin C & E, zinc, saffron) in neovascular AMD demonstrated improvements in visual acuity and partial restoration of gut microbial diversity and metabolite profiles, supporting the concept that dietary interventions may modulate the gut–retina axis [80]. Furthermore, large cohort studies consistently demonstrate Mediterranean-style diets rich in fruits, vegetables, legumes, whole grains, and fish has been associated with up to a 41% reduced risk of AMD progression [77, 81]. This provides high levels of antioxidants and diverse plant polyphenols, which fosters a gut microbiome that produces anti-inflammatory SCFAs and maintains intestinal integrity [27, 82].

The direct modulation of gut flora is also a promising method to reduce disease severity (Figure 2). Mouse models have demonstrated that aged microbiota are enriched with *Prevotella, Lachnospiraceae,* and *Faecalibaculum* species alongside a depletion of long-chain fatty acid synthesis whereas young microbiota had *greater Bifidobacteria, Eubacteria,* and *Akkermansia* species. Parker et al. showed that FMT from old mice into young recipients accelerated retinal inflammation and microglial activation, whereas transplantation of a young microbiome into aged mice reduced retinal inflammatory markers and preserved retinal function [83]. Broadfield et al. demonstrated that in mice with syngeneic colon adenocarcinomas, FMT from metformin-treated mice to high fat diet-mice increased the expression of SCFA-producing microbes and reduced tumor cell growth [84]. Future studies exploring the use of FMT or nutrient supplementation may provide novel approaches to reducing AMD risk and progression.

Beyond the microbiome, drug repurposing strategies may also hold promise for treatment of AMD through microbiome-mediated pathways without undergoing the costs and approval processes associated with traditional drug development (Figure 2) [85]. Metformin, for example, has been associated with reduced AMD incidence and slower geographic atrophy progression, and is known to alter gut microbial composition and increase SCFA-producing taxa in metabolic disease settings [86–89]. Statins and doxycycline are also known to alter the gut microbiome and have independently shown promise of modifying AMD risk, however these effects remains limited with mixed epidemiologic findings and predominantly non-microbiome mechanistic data [90–93]. Future work should disentangle whether observed ocular benefits of these agents reflect direct retinal actions, microbiome modulation, or both before they can be considered true gut–retina therapies.

Despite these encouraging preclinical and early clinical observations, important limitations and knowledge gaps remain. Most human microbiome studies in AMD are cross-sectional, rely on small sample sizes, and are often confounded by age, diet, medications, and comorbidities, making it difficult to infer causality or define disease-specific microbial signatures. Longitudinal cohorts with standardized dietary assessment, multi-omic profiling, and careful phenotyping across AMD stages are needed to clarify temporal relationships between dysbiosis and retinal degeneration. Additionally, as most current data derive from animal models, mechanistic work is still required to clarify how specific taxa, metabolites, and host genetic variants converge on complement activation, inflammasome signaling, and microglial responses in the human retina.

## Discussion

The gut-retina axis provides a framework for understanding how systemic dysbiosis influences AMD pathogenesis. Altered gut microbial communities and reduced production of metabolites such as SCFAs and BAs contribute to chronic inflammation, complement dysregulation, and angiogenesis in the retina. These effects are modulated by genetic risk factors and compounded by high-fat/high-glycemic dietary patterns. Dietary modifications, FMTs, and drug repurposing strategies may offer strategies for enhancing beneficial metabolite production and prevent the risk of progression to advanced disease stages. However, current evidence is largely based on cross sectional microbiome profiling and preclinical models, so causal pathways between gut dysbiosis and AMD progression remain incompletely defined. Future work incorporating longitudinal human cohorts and microbiome targeted interventions will be essential to address these gaps and determine whether modulating the gut–retina axis can meaningfully alter visual outcomes in AMD.
